# Analysis of Factors Contributing to the Injury Severity of Overloaded-Truck-Related Crashes on Mountainous Highways in China

**DOI:** 10.3390/ijerph19074244

**Published:** 2022-04-02

**Authors:** Huiying Wen, Yingxin Du, Zheng Chen, Sheng Zhao

**Affiliations:** School of Civil Engineering and Transportation, South China University of Technology, Guangzhou 510641, China; hywen@scut.edu.cn (H.W.); yxdu24@163.com (Y.D.); zhengchenscut@163.com (Z.C.)

**Keywords:** injury severity, overloaded-truck-related crashes, random parameter logit model, mountainous highways, unobserved heterogeneity

## Abstract

Overloaded transport can certainly improve transportation efficiency and reduce operating costs. Nevertheless, several negative consequences are associated with this illegal activity, including road subsidence, bridge collapse, and serious casualties caused by accidents. Given the complexity and variability of mountainous highways, this study examines 1862 overloaded-truck-related crashes that happened in Yunnan Province, China, and attempts to analyze the key factors contributing to the injury severity. This is the first time that the injury severity has been studied from the perspective of crashes involving overloaded trucks, and meanwhile in a scenario of mountainous highways. For in-depth analysis, three models are developed, including a binary logit model, a random parameter logit model, and a classification and regression tree, but the results show that the random parameter logit model outperforms the other two. In the best-performing model, a total of fifteen variables are found to be significant at the 99% confidence level, including random variables such as freeway, broadside hitting, impaired braking performance, spring, and evening. In regards to the fixed variables, it is likely that the single curve, rollover, autumn, and winter variables will increase the probability of fatalities, whereas the provincial highway, country road, urban road, cement, wet, and head-on variables will decrease the likelihood of death. Our findings are useful for industry-related departments in formulating and implementing corresponding countermeasures, such as strengthening the inspection of commercial trucks, increasing the penalties for overloaded trucks, and installing certain protective equipment and facilities on crash-prone sections.

## 1. Introduction

Over the years, road safety issues have attracted great attention from all walks of life. Although the industry management departments in various countries have taken proper measures, and meanwhile achieved certain results, the annual number of road traffic deaths remains unacceptably high, reaching 1.35 million in 2016 [1]. As we all know, there are a number of factors that can contribute to road traffic accidents, among which the inappropriate transportation behavior of commercial trucks, especially truck overloading, is one of the main causes. In a narrow sense, truck overloading is the act of loading a commercial truck with more cargo than the vehicle’s rating [2]. This phenomenon is quite common in middle-income countries, where the demand for cargos transportation is relatively considerable. Undeniably, overloaded transportation does help enterprises improve their transportation efficiency and reduce operating costs, but at the same time, it may also pose serious potential risks to road safety, and can even lead to severe accidents. Consider the case of China. According to statistics, more than 80% of truck-related crashes result from truck overloading [3]. In addition, compared with properly loaded vehicles, overloaded trucks are far more likely to damage road traffic infrastructure, as manifested in shortening the service life of the road pavement, increasing the fatigue damage of the bridge, and even causing the bridge to collapse [4,5]. Truck overloading is also associated with other negative phenomena, including the disorder of the road transport market and an increase in maintenance costs for hardware infrastructure [6,7]. Considering the prevalence and hazards of truck overloading, it is of great practical significance to conduct appropriate research and analysis on overloaded trucks to improve road safety.

On the whole, however, there are few studies that are specific to overloaded trucks, and the existing related works are more focused on the dangers of truck overloading [4,5,8,9,10]. In terms of the injury severity of truck-related crashes, studies oriented to such vehicles are even less common but may include factors such as the size of the load or whether the vehicle is overloaded as an element to examine [11,12]. Unlike previous studies, this paper focuses only on overloaded-truck-related crashes and analyzes the key factors affecting the injury severity of those crashes, based on five aspects: roadway characteristics, environmental characteristics, accident characteristics, vehicle characteristics, and temporal characteristics. Despite the five characteristics mentioned above having been considered more in established studies, this paper proposes some new elements in combination with the categorization features of vehicles and roads in China, thus refining the values of the variables. On mountainous highways, the driving environment is more complicated, with long downward slopes, tunnels and even tunnel groups on some sections [13]. Additionally, this type of road is commonly used in China for the transportation of minerals or construction materials. Considering the two facts above, commercial trucks, especially overloaded vehicles, may be at greater risk in such an environment. In light of this, as opposed to most prior studies that have chosen plain areas as the scenario, this paper is more interested in the crashes that occur in mountainous regions. Analysis results are of great value for developing strategies for the management of overloaded trucks and the improvement of road safety on mountainous highways.

Numerous works have demonstrated that logit models are effective tools for exploring the factors that influence the injury severity of truck-related crashes [14,15,16,17,18,19]. To address the heterogeneity among drivers, this paper also introduces random parameters to fit the crash data. Further, the fitting performances between different models are compared, mainly considering the traditional logit model and the classification and regression tree method. The remaining sections in this paper are organized as follows. Next, related works on the hazards of overloaded trucks and the influencing factors of truck-related crashes are summarized. In the following section, data description is presented, and the modeling process is described. As for Section 4, the estimated results of the models are listed, and the corresponding analysis and discussion follows. A summary of the whole study and a vision for future studies are stated in the last section.

## 2. Literature Review

Currently, studies on overloaded trucks tend to focus on their negative effects, such as destruction of road traffic infrastructure, interference with social order and damage to the living environment [4,5,6,7,8,9,10]. Jacob et al., have confirmed that overloaded trucks can contribute to a significant reduction in the service life of road pavements [8]. Another study exploring the effect of overloaded trucks on the surrounding environment has shown that overloaded vehicles, especially trucks with trailers and semi-trailers, tend to generate more noise than other types [9]. In addition, some scholars have described the application practice of the weight-in-motion (WIM) technology, a system that measures a vehicle’s weight while it is traveling normally or at reduced speeds, and has attempted to perform prediction or analysis based on the historical data [20,21,22]. Unfortunately, there is still a large research gap in the analysis of overloaded-truck-related crashes, particularly in the exploration of factors that contribute to the injury severity.

For all types of truck-related crash, dozens of characteristics have been explored in the previous studies up to now, involving multiple scenarios and covering various perspectives. In the selection of regions studied, both developed and developing countries have been involved, especially the latter, whose road traffic deaths tend to take a larger proportion [23]. Specific to the selection of traffic scenarios, rural and urban roadways [24], mountainous and non-mountainous highways [12,13,19], freeway merging and diverging points [25], roadway sections and signalized intersections [26], or other typical locations such as toll plazas [26], river-crossing tunnels [27], work zones [28] and even highway-rail grade crossings [29] have been considered. With respect to mountainous highway scenarios, Wang et al., examined the influence of road geometric design on the injury severity of truck-related crashes, using two typical freeways in China, and found that curve factors significantly increased the likelihood of fatalities, but the opposite was true in the case of downgrade sections [12]. Similarly, Zhou et al. selected freeways in a mountainous region of China as the scenario and compared the differences in the injury severity between tunnel sections and general sections [30]. Based on the estimated results, it appears that trucks were involved in more severe crashes in tunnel sections, and the risk of drivers or occupants being fatally injured is higher in the long tunnels that range from 1000 m to 3000 m in length.

Likewise, in terms of influencing factors selection, a considerable number of characteristics have been extensively studied, mainly including roadway characteristics, driver characteristics, environmental characteristics, accident characteristics, vehicle characteristics and temporal characteristics [19,31,32]. The weight of the vehicle or whether the truck is overloaded should be one of the elements in the above-mentioned vehicle characteristic group, which normally includes factors such as truck type, braking performance and tire condition [12,16,18]. As expected, truck overloading has been shown in several studies to result in more severe injury consequences [12,27,33]. A study conducted by Chen et al. found that higher casualty levels were associated with overloaded trucks and inferred that the outcome was related to an increase in braking distance [27]. Focusing on the vehicle weight factor, Li et al., have demonstrated that higher values are associated with greater levels of accident injuries and fatalities on normal sections of highways, and they have emphasized the importance of designing and installing emergency escape ramps for heavy trucks, especially the truck overloaded [34]. Considering the negative impact of truck overloading or increased weight on the injury severity, there is a strong need for a separate detailed study on overloaded-truck-related crashes so as to truly identify the key elements or scenarios that may lead to a high probability of fatalities.

Among the other five categories, driver characteristics, especially driving behavior, is identified as the most significant aspect that affects the injury severity of truck-related crashes [13]. Risky driving behaviors such as disregarding traffic signs or signals, speeding, drunk or drugged driving, fatigued driving and distracted driving have been found to induce more severe injury outcomes, while safe driving behaviors, for example, wearing a seat belt, have been shown to decrease the severity [29,35,36]. Other factors falling under the driver characteristic group, such as age, gender, alcohol influence, and drug involvement, also play a role in determining crash occurrence and injury severity [26,37,38]. Regarding the selection of factors related to roadway characteristics, horizontal and vertical alignment, curve radius, pavement surface conditions, the number of lanes and speed limits were generally included [24,25,31,39,40]. Concerning the selection of factors related to environmental characteristics, weather conditions, lighting conditions and traffic volume were often covered [29,39,41]. With regard to the selection of factors related to accident characteristics, crash type and the number of vehicles involved were considered in most cases [37,42]. As for the selection of factors related to temporal characteristics, time of day, day of week and season were included to a great extent [15,33,38,43].

Methodologically, logit and probit models are the most widely employed methods for analyzing the injury severity of truck-related crashes [13]. A number of the logit models used in previous studies have been modified in order to provide a better fit to the data. The random parameter logit model, for example, is obtained by adding a random term to the original model. In addition, mainstream hybrid models, like the Bayesian logit model, are developed by incorporating appropriate theoretical methods into the basic model [14,15,16,17,18,19]. Apart from traditional regression models, more and more new methods have been applied to injury severity analysis in recent years, such as the partial proportional odds model (PPO) [12], classification and regression tree (CART) [13,35] and latent class clustering (LCC) [36].

Based on the literature review discussed above, this study decides to center on the overloaded-truck-related crashes that occurred on the mountainous highways in Yunnan Province, China. Meanwhile, given that the random parameter logit model can account for the unobserved heterogeneity, which meets our study objectives, it is chosen in this paper, as in several others, to analyze the injury severity of those crashes [15,19,41,44,45]. It is expected that this study will help clarify the key elements affecting the injury severity, which is of great value for both research gaps filling and road safety governance.

## 3. Materials and Methods

### 3.1. Data

Over 3000 truck-related crashes are utilized in this study, which occurred in Yunnan Province between 2010 and 2015, collected by the local traffic police departments and crash appraisal departments. In the original dataset, there are a number of pre- and post-crash data items. In the pre-crash group, roadway characteristics (e.g., road grade, horizontal alignment, and pavement condition), truck characteristics (e.g., vehicle performance status and truck type), and environmental characteristics (e.g., lighting condition) are involved, while in the post-crash category, accident characteristics (e.g., collision type and casualty figure) and temporal characteristics (e.g., the date of accidents) are recorded. Other characteristics such as seat belt usage and airbag status are also investigated and written into the dataset.

However, the collected data is inevitably flawed due to possible errors in manual data entry. Specifically, there are instances where data is missing or inconsistent. In this regard, prior to data analysis, the existing data need to be properly processed, which mainly involves three aspects in our study: data repair, data filtering and data regeneration. Thereafter, five crash characteristic groups are re-formed, including roadway characteristics (i.e., highway classification, horizontal alignment, pavement material, and road surface condition), environmental characteristics (i.e., lighting condition), accident characteristics (i.e., collision type), vehicle characteristics (i.e., truck type and braking performance) and temporal characteristics (i.e., season and time). Those data items not included in the revised dataset are used as reference items, so as to provide certain factual support for the inference. As for the selection of injury severity indicators, the KABCO scale, which has been frequently used in previous studies, is referenced [40,44]. Nevertheless, as few records of non-injury outcomes are written into the original dataset, and as casualties are described in a relatively simple manner, only the indicators of death and injury (both apparent and non-apparent) are considered. Ultimately, a total of 1862 overloaded-truck-related crashes are included in the study sample. A brief description of the data characteristics of the explanatory variables can be found in Table 1.

As shown in Table 1, there were 1160 overloaded-truck-related crashes that resulted in more severe consequences, accounting for 62.30% of the total, which is 1.65 times the number of crashes that resulted in only injuries. Regarding the explanatory variables, more crashes occurred in the following scenarios: country road, straight section, asphalt pavement, dry surface, daylight, head-on collision, light truck, normal braking performance, winter, and afternoon. Contrary to intuitive thinking, crashes that occurred on dry roads accounted for the majority of the sample size, taking up 91.62% of it, and more than 60% of those involved more severe outcomes. An accurate and detailed analysis must be conducted through modeling.

### 3.2. Methodology

Due to the heterogeneity of truck drivers, a random parameter logit model was developed for this problem. In addition, in order to demonstrate the superiority of the stochastic model, a traditional binary logit model, as well as a classification and regression tree model, were used as references. The construction process of the above three models is presented below.

#### 3.2.1. Binary Logit Model

As mentioned earlier, only two injury severity indicators, death and injury, were selected. Thus, whether an overloaded truck will cause fatalities in the crash can be approximately regarded as a binary choice problem. In order to solve this problem, a simple linear probability model is often considered [46]. However, due to the difficulty that the predicted value calculated by the model can be guaranteed to always fall within a closed interval $\left[0,1\right]$, and the heteroscedasticity of the disturbance term, it is necessary to transform the linear probability model into a utility model [47]. On this basis, it is also essential to select an appropriate probability distribution for the disturbance term according to practical needs [48].

In general, both the logistic distribution and the standard normal distribution have higher usage rates; the former can be used to obtain a logit model, while the latter can be used to derive a probit model. Equation (1) shows the specific formula of a binary logit model [14,49,50,51]:

(1) $$F(X_{i}B)=\exp(X_{i}B)/[1+\exp(X_{i}B)]$$

where $X_{i}$ is a vector of several explanatory variables; B is a vector of the coefficients to be estimated, matching with the vector $X_{i}$; F(z) is the cumulative distribution function of that disturbance term; n is the total number of observations (i.e., the crashes); i is the unique number of each observation, ranging from 1 to n. Correspondingly, the marginal effects of those explanatory variables can be calculated by Equation (2) [44,52,53]:

(2) $$\partial F(X_{i}B)/\partial x_{j}=\beta_{j}f(X_{i}B)$$

where $x_{j}$ is an arbitrary variable; $\beta_{j}$ is the coefficient of a certain variable, f(z) is the corresponding probability density function; m is the total number of variables added to the model; j is the unique number of each variable, ranging from 1 to m.

#### 3.2.2. Random Parameter Logit Model

In the traditional binary logit model, the coefficient of each explanatory variable is assumed to be fixed; that is to say, one plays an equal role in all crashes [44]. However, this is not exactly the same as in reality. For different drivers, there exist more or fewer discrepancies in their characteristics such as driving habits, behavior preference and risk awareness. Thus, despite the same scenario, different drivers may make different decisions, resulting in different levels of consequences. It is in consideration of the unobserved heterogeneity problem that random parameters are introduced.

Unlike the assumption of the previous model, the random parameter logit model assumes that the corresponding coefficients of the explanatory variables can obey a certain distribution. Referring to the existing studies, the random parameter is also assumed to be normally distributed in our study, and to be written as a linear combination of a fixed parameter and a random term, as shown in Equation (3) [15,41,44,54,55,56]:

(3) $$\beta_{i}=\beta +\mu_{i}$$

where $\beta_{i}$ is the coefficient of the variable for observation i considering the random utility, and $\mu_{i}$ is a normally distributed term corresponding to that observation. Combined with Equation (1), a random parameter logit model can be obtained, as shown in Equation (4):

(4) $$F(X_{i}B_{i})=\exp(X_{i}B_{i})/[1+\exp(X_{i}B_{i})]$$

where $B_{i}$ is a vector of those random coefficients corresponding to observation i.

The estimation of random parameters usually requires simulation, mainly involving the Maximum Likelihood Estimate and the Halton Sequence [41,43]. In general, 200 Halton draws are often utilized [19,39,41,44,45], while in this study, the sampling frequency is increased to 500, so as to improve the accuracy of the estimated results.

#### 3.2.3. Classification and Regression Tree

In machine learning, a Decision Tree (DT) is a supervised learning method, while a Classification and Regression Tree (CART) is one of the algorithms under this category, either as a non-parametric classification method or as a non-parametric regression one [13]. When the dependent variable is continuous, the generated tree is a regression tree, but when it is discrete, a classification tree is generated. The major difference between CART and other algorithms under DT is that the tree built by CART is necessarily a binary tree [35,57]. In other words, no matter what type of decision tree is used, or how many values the characteristic variables have, each node can only be split into two child nodes based on the relationship between the value of the variable and the discriminant rule, where the one that satisfies the rule is “yes” and the one that does not is “no”. In general, the left branch of the tree generated corresponds to the branch with “yes” feedback, while the right branch corresponds to the one with “no” feedback. By recursively splitting each characteristic node according to such rules until there are no further splits that can be made, a decision tree based on CART can be obtained [13,35]. Based on the tree-building process presented above, it can be concluded that the CART algorithm is relatively simple compared with the previous logistic regression models [20,57]. Since the dependent variable in this study is a discrete one that is either death or injury, the decision tree generated using CART is a classification tree. Subsequently, it is sometimes necessary to prune the trees based on the classification results, as well as to select the optimal tree [13,35,57].

## 4. Results and Discussion

**Figure 1:**
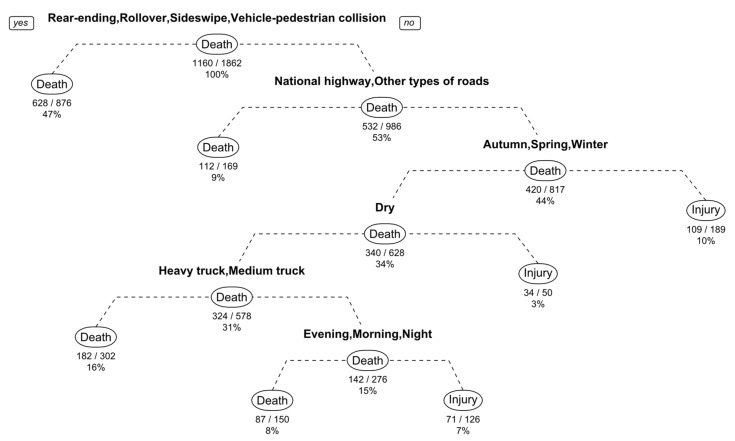
Decision tree built by CART. Note. a/b = the number of “yes” feedback/the number of observations.

Based on the revised dataset, a traditional binary logit model, a random parameter logit model, and a decision tree based on CART are developed, whose estimated results are shown in Table 2 and Figure 1.

### 4.1. Model Comparison

#### 4.1.1. Binary Logit Model vs. Random Parameter Logit Model

Table 2 demonstrates that there are certain differences between the estimated results of these two models, firstly reflected in the number of significant variables. In the traditional binary logit model, a total of nine variables are significant at the 99% confidence level, while in the random parameter logit model, the number of significant variables rises to 15, five of which are found to be random.

In order to evaluate the estimation effect of the two models more accurately, and to compare their goodness-of-fit, Log-likelihood Function and Akaike Information Criterion (AIC) are introduced. According to the results shown in Table 2, the model including random parameters has a greater value of Log-likelihood at convergence and a smaller value of AIC, compared with the traditional one. Theoretically, for models that utilize the same dataset, one with a greater Log-likelihood at convergence and a smaller AIC is considered to be superior [41]. Therefore, it can be concluded that the random parameter logit model outperforms the traditional model.

On this basis, this study also attempts to determine the difference between those two models by utilizing a likelihood ratio test. The null hypothesis of the test is that there is no statistical difference between the binary logit model and the one with random parameters. Subsequently, Equation (5) can be used to calculate the corresponding Chi-square statistic value [58]:

(5) $$\chi^{2}=-2(LL_{\beta }{}_{{}_{Traditional}}-LL_{\beta }{}_{{}_{Random}})$$

(6) $$\chi^{2}=-2[-1151.216-(-1144.624)]=13.184$$

where $\chi^{2}$ is the test statistic; $LL_{\beta }{}_{{}_{Traditional}}$ is the Log-likelihood at the convergence of the binary logit model; and $LL_{\beta }{}_{{}_{Random}}$ is the Log-likelihood at the convergence of the random parameter logit model. In light of the above Chi-square statistic value and the corresponding degree of freedoms, 5 (the number of random parameters), it can be found that the calculated value (13.184) is greater than the critical value (12.833) at the 97% confidence level. Consequently, the aforementioned null hypothesis is rejected, which again proves that the random parameter logit model is superior to the traditional one.

#### 4.1.2. Binary Logit Model vs. Classification and Regression Tree Model

Similarly, there are also several differences between the results obtained by these two models with respect to the discrimination of influencing factors, which are mainly reflected in the categories of the factors. In the binary logit model, a total of eight categories of influencing factors are significant at the 99% confidence level, while the number of major influencing factors in the decision tree model is only 6. Furthermore, the factors included in these two models also differ to some extent. Specifically, horizontal alignment, pavement material and braking performance are factors that are unique to the binary logit model, while the factor of truck type is unique to the CART model.

However, unlike the comparison between the logistic regression models, in the field of machine learning, the prediction accuracy of different algorithms is often compared using the ROC curve and the AUC value [59]. In the ROC curve image, the horizontal axis is typically labeled with the false positive rate (FPR), the proportion of samples incorrectly judged as positive among all negative samples, while the vertical one is labeled by the true positive rate (TPR), the percentage of samples correctly interpreted as positive among all positive samples [59,60,61,62,63]. Theoretically, the model with lower FPR and higher TPR is a more ideal model. Therefore, the more skewed the ROC curve is to the upper left, the more effective the algorithm is. Correspondingly, the point (0,1) represents the optimal model, in which all samples are correctly classified [59,60,61].

Based on the ROC curve, a statistical evaluation metric of the AUC value is proposed in order to quantitatively characterize the classification capability of the algorithm. The initialism “AUC” stands for “area under the curve”, specifically, the ROC curve, and is measured between 0 and 1 [59,61,62]. When AUC equals 1, it means that the model is a perfect classifier, but this is rarely the case. When the value of AUC is between 0.5 and 1, it denotes that the model is superior to random guessing. When AUC equals 0.5, it indicates that the model is as effective as random guessing [59,61]. Obviously, it is more accurate and straightforward to compare the prediction effect of different algorithms by means of AUC. Therefore, the evaluation index of AUC was used for this study to compare the excellence of the binary logit model with the classification and regression tree model. The calculated results show that the AUC value is 0.631 for the binary logit model, while the value of CART is only 0.579, which is lower than the former. Consequently, it can be concluded that the binary logit model outperforms CART in terms of prediction.

Combining the comparison result of the two logit models, it is also possible to conclude that the random parameter logit model is superior to CART as the most suitable model. Thus, the subsequent analysis is conducted based only on the estimated results obtained from the random parameter logit model.

### 4.2. Detailed Analysis and Discussion

Table 3 shows the marginal effects of the logit model with random parameters. As those explanatory variables that are significant at the 99% confidence level only occur in four characteristic groups (roadway characteristics, accident characteristics, vehicle characteristics, and temporal characteristics), the detailed analysis and discussion are limited to these categories.

#### 4.2.1. Roadway Characteristics

In the roadway characteristic group, a total of seven variables are significant at the 99% confidence level, including freeway, provincial highway, country road, urban road, single curve, cement, and wet. However, only the freeway variable is found to be random in the model and to be normally distributed with a mean of −0.733 and a standard deviation of 0.900. It can be calculated that 79.1% of overloaded-truck-related crashes occurring on freeways are less than 0, while 20.9% of these crashes are greater than 0. In other words, 79.1% of overloaded-truck-related crashes that took place on freeways are less likely to cause deaths, whereas 20.9% of these crashes increase the probability of severe consequences. As compared to other types of roads, freeways in China have a relatively simple traffic composition. Most freeways are used exclusively by motor vehicles, with very few motorcycles, non-motorized vehicles, or pedestrians [64]. As a result of this, direct collisions between trucks and human bodies are extremely unlikely, and fatalities are more likely to result from vehicle–vehicle crashes. Given the predominance of rear-end and sideswipe collisions in freeway crashes, and the relatively minor injury severity in these collisions [41,65], drivers or occupants are more likely to suffer only injuries on freeways. However, several existing studies have confirmed that high-speed limits may increase the probability of severe crashes [31,49,66]. As overloaded trucks have a longer braking distance than other vehicles, there is still a risk that the truck will collide at a higher speed and cause fatalities when the distance between the preceding and following vehicles is not kept properly [27].

As for the remaining six variables, all coefficients are found to be fixed. However, only the single curve variable increases the probability of death, with a marginal effect of 0.089. As everyone knows, the visibility of mountainous highways is poor, especially at the bends of the two-lane winding mountain roads. Hence, it is quite challenging for drivers to detect oncoming vehicles in advance and take proper measures in time [66]. Drivers are thus more likely to experience collisions on single-curve sections with more severe outcomes than on straight sections.

On the contrary, the provincial highway, country road, and urban road variables tend to reduce the likelihood of death, with marginal effects of −0.117, −0.140, and −0.110, respectively. In principle, the lower functional grade of highways or roads, compared with national highways and mainly embodied in the narrow lane width or the small number of lanes, limits the speed of vehicles. In low-speed operation, the impact generated by the crash is relatively small, consequently, with lower odds of resulting in severe outcomes [29,67]. Besides, according to a previous study, the lower driving speed may also be related to the changeable alignment of mountainous highways [12]. Additionally, the cement and wet variables can likewise result in minor outcomes, with marginal effects of −0.082 and −0.105, respectively. In terms of causes, the former may be due to the anti-skid characteristics of cement pavements, while the latter, slightly different from the intuitive opinion, may be due to the higher risk awareness of drivers [11,32,40].

#### 4.2.2. Accident Characteristics

At the 99% confidence level, a total of three variables in the accident characteristic group are significant, which are head-on, broadside and rollover. However, only the broadside variable is found to be random, and to obey a normal distribution, with a mean of −0.878 and a standard deviation of 1.472. It can be calculated that 72.6% of overloaded-truck-related crashes whose collision type is broadside hitting are less than 0, while 27.4% of these crashes are greater than 0. In other words, among the overloaded-truck-related crashes with a broadside hitting characteristic, 72.6% of those are less likely to result in severe consequences, whereas less than one-third of the crashes may increase the likelihood of death. Intuitively, that negative effect seems reasonable because the vehicle body, especially the body of the truck, can shield the driver or occupants from some of the impact generated by the broadside hitting [44,65]. In multi-truck crashes, the impacted part is usually far from the cab, so the odds of fatal injuries to the driver or occupants are also reduced. However, in certain scenarios, broadside hitting may also have severe consequences, closely related to the driver’s reckless driving behavior and poor risk awareness [15,45]. An example would be a truck traveling at a high speed through an unsignalized intersection and colliding with a car.

As for the head-on and rollover variables, both coefficients are found to be fixed, but one is negative and the other is positive, with marginal effects of −0.135 and 0.156, respectively. In truck-car crashes, due to the massive size of the overloaded truck, as well as the effective role played by the energy absorption equipment, which is installed in the front of the car, the consequences of a head-on collision between these two types of vehicles are relatively less severe [68]. For truck–truck crashes, however, this finding is not in agreement with the established research results [65]. It is speculated that the negative impact of a head-on collision is more related to the slower driving speed of trucks that are overloaded. In rollover accidents, the vehicle’s center of gravity is raised as a result of overloading, and drivers or occupants inside the truck are at a high risk of fatal injury, especially if the seat belt is not worn properly [24,32,33,49]. Further, if an overloaded truck is involved in a rollover accident on the winding mountain road section, and meanwhile runs off the road, the huge drop between the cliff and the valley may result in more severe injuries.

#### 4.2.3. Vehicle Characteristics

In the vehicle characteristic group, only the impaired variable, which refers to diminished or failed braking performance, is found to be significant and random at the 99% confidence level. Based on the estimated results, the variable is normally distributed with a mean of 0.617 and a standard deviation of 0.657. Correspondingly, 17.4% of crashes involving overloaded trucks with impaired braking performance are less than 0, while 82.6% of these crashes are greater than 0. In other words, 17.4% of crashes caused by overloaded trucks whose braking performance is impaired are less likely to result in a fatality, whereas more than four-fifths of crashes increase the likelihood that someone would be fatally injured or even dead. The positive effect of damaged braking systems on a vehicle would appear to be reasonable. Combining the effects of truck overloading and impaired braking performance, the braking distance of overloaded trucks is greatly increased [27]. If the system fails completely, it is more likely that the vehicle will collide at its original or even higher speed. As a result, the impact of the crash on drivers or occupants may be greatly increased, and accordingly, increase the probability of death. However, because part of the fact that mountainous highways’ long downhill sections, for example, have been set up with emergency escape ramps, vehicles with impaired braking performance can reduce their speed. It is therefore possible for a few overloaded trucks to cause only injuries in a crash, especially in a single-vehicle collision [34].

#### 4.2.4. Temporal Characteristics

In the temporal characteristic group, a total of four variables are significant at the 99% confidence level, including spring, autumn, winter and evening. However, two of these variables, autumn and winter, are found to be fixed, with marginal effects of 0.072 and 0.094, respectively. That is to say, in comparison with the overloaded-truck-related crashes that occur in summer, those occurring in autumn and winter are more likely to result in fatalities. In an analysis of the historical weather records of Yunnan Province, it has been found that foggy days are most common during the winter, accounting for 40% of the total number of foggy days in a year, while autumn comes in second place [69]. In general, the presence of dense fog or agglomerate fog in mountainous areas can greatly reduce visibility, making it difficult for drivers to identify the road conditions ahead. Whenever there is a small radius curve, and the driver fails to take proper measures in time, the truck is very likely to run off the road or even fall off the cliff, resulting in severe outcomes [12].

In contrast, the remaining two variables, spring and evening, are found to be random in the model. For the spring variable, it obeys a normal distribution, with a mean of 0.556 and a standard deviation of 2.538. It can be calculated that 41.3% of overloaded-truck-related crashes occurring in spring are less than 0, while 58.7% of these crashes are greater than 0. In other words, 41.3% of overloaded-truck-related crashes that took place in spring are less likely to cause deaths, whereas 58.7% of these crashes increase the probability of severe consequences. Due to the typically dry climate, with relatively little precipitation, in most parts of Yunnan Province during spring, it is likely that this finding relates more to the driving habits of overloaded truck drivers [69]. Drivers who underestimate driving risks and break traffic rules, especially when speeding, often fail to take timely braking measures, thus increasing the probability of severe consequences [33,36]. On the other hand, those who adhere to the rules and remain cautious will greatly reduce their chances of severe consequences.

As for the evening variable, it is also normally distributed with a mean of 0.873 and a standard deviation of 3.369. It can be calculated that 39.7% of overloaded-truck-related crashes occurring in the evening are less than 0, while 60.3% of these crashes are greater than 0. In other words, among the overloaded-truck-related crashes that occurred in the evening, 40.1% of those are less likely to result in severe consequences, whereas about three-fifths of the crashes may increase the injury severity. One possible explanation for this positive impact is the poor visibility in the evening, which drastically reduces the driver’s ability to identify risks. In the event of an abnormal situation on the road, it is less likely that the driver will be able to react quickly and correctly, possibly resulting in a high-speed crash [29,38,43]. This is particularly true for those overloaded trucks with poor braking performance. Several studies have shown that high-speed crashes tend to increase the injury severity, and thus an overloaded truck that collides in the evening may bring about more serious consequences [13,70]. This analysis can also be applied to fatigued driving. In contrast, when a driver is not fatigued but is attentive to his surroundings, the likelihood of a serious crash will be reduced to some extent.

## 5. Conclusions

Overloaded transportation can indeed bring substantial economic profits to transport enterprises. However, various consequences resulting from this illegal activity, such as road subsidence, bridge collapse, as well as serious casualties in the event of accidents, should not be overlooked. Considering the prevalence and seriousness of truck overloading, this study focused on the overloaded-truck-related crashes that occurred on mountainous highways, utilizes the truck crash data in Yunnan Province between 2010 to 2015, and developed a random parameter logit model to analyze the impacts of five types of characteristics (i.e., roadway characteristics, environmental characteristics, accident characteristics, vehicle characteristics, and temporal characteristics). As far as we know, this is the first attempt at exploring factors that contribute to the injury severity of crashes from the perspective of overloaded trucks. In addition, based on the characteristics of the road system in China, this study provided a more detailed categorization of the highway classification factor, which has never been attempted before. A similar process was also used to categorize the truck type factor.

Based on the estimated results, the following conclusions can be drawn. First, the binary logit model considering random parameters outperformed the traditional one as well as CART, which is closely related to the model’s ability to account for part of the unobserved heterogeneity. Second, at the 99% confidence level, a total of fifteen variables were found to be significant in the random parameter logit model. These variables covered four characteristic groups (i.e., roadway characteristics, accident characteristics, vehicle characteristics, and temporal characteristics), but only five of them were found to be random, which are freeway, broadside hitting, impaired braking performance, spring, and evening. For those fixed variables, single curve, rollover, autumn, and winter were likely to increase the probability of severe consequences, while the provincial highway, country road, urban road, cement, wet, and head-on variables generally reduced the likelihood of death. In terms of the random variables, more overloaded-truck-related crashes that occurred in spring or the evening may increase the probability of fatalities. There was a similar pattern in crashes involving overloaded trucks with impaired braking performance. However, the findings were just the opposite in the scenario of freeway or broadside hitting.

In the face of those severe outcomes resulting from overloaded-truck-related crashes, proper countermeasures should be proposed and implemented as soon as possible. For example, related laws and regulations should be continually improved, so as to provide strong support for strengthening the supervision of transport enterprises and punishing those operators involved in truck overloading. On this basis, more checkpoints should be set up at the entrances and exits of cities and towns, as well as on those accident-prone sections, in order to verify the actual load of trucks. It is also recommended to install more protective facilities or equipment, for example, emergency escape ramps, on mountainous highways to create safer driving conditions for drivers. Limited by the quality of our dataset, only a small number of factors are available for analysis, and thus the study perspective is still not comprehensive enough. In this regard, further studies may consider accessing other datasets to add new factors. Additionally, the five random variables obtained in this study were estimated based on the selected dataset and passed the significance test. However, it is still possible that other random variables remain uncovered as a result of missing data. Further studies may consider replacing another dataset for validation. Furthermore, other suitable methods can be applied to the injury severity analysis of overloaded-truck-related crashes, in the hope of continuously improving the accuracy of the estimates.

## Figures and Tables

**Table 1:** Statistical characteristics of explanatory variables.

Variables\Indices	Death		Injury		Total	
	Frequency	Percentage	Frequency	Percentage	Frequency	Percentage
*Total crashes*	1160	62.30%	702	37.70%	1862	100.0%
*Roadway characteristics*						
*Highway classification*						
Freeway	207	65.30%	110	34.70%	317	17.02%
National highway *	182	72.22%	70	27.78%	252	13.53%
Provincial highway	124	59.33%	85	40.67%	209	11.22%
Country road	396	58.41%	282	41.59%	678	36.41%
Urban road	144	57.83%	105	42.17%	249	13.37%
Urban expressway	11	55.00%	9	45.00%	20	1.07%
Other types of roads	96	70.07%	41	29.93%	137	7.36%
*Horizontal alignment*						
Straight *	935	61.31%	590	38.69%	1525	81.90%
Single curve	221	67.38%	107	32.62%	328	17.62%
Consecutive curve	4	44.44%	5	55.56%	9	0.48%
*Pavement material*						
Asphalt *	932	63.32%	540	36.68%	1472	79.05%
Gravel	41	74.55%	14	25.45%	55	2.95%
Cement	164	52.90%	146	47.10%	310	16.65%
Dirt	23	92.00%	2	8.00%	25	1.34%
*Road surface condition*						
Dry *	1077	63.13%	629	36.87%	1706	91.62%
Wet	78	52.00%	72	48.00%	150	8.06%
Other types of conditions	5	83.33%	1	16.67%	6	0.32%
*Environmental characteristics*						
*Lighting condition*						
Daylight	772	61.27%	488	38.73%	1260	67.67%
Dark lit *	315	66.60%	158	33.40%	473	25.40%
Dark, not lit	73	56.59%	56	43.41%	129	6.93%
*Accident characteristics*						
*Collision type*						
Head-on	375	54.82%	309	45.18%	684	36.73%
Broadside	106	48.18%	114	51.82%	220	11.82%
Rear-ending	197	67.47%	95	32.53%	292	15.68%
Sideswipe	77	66.96%	38	33.04%	115	6.18%
Vehicle–pedestrian collision *	206	69.59%	90	30.41%	296	15.90%
Rollover	148	85.55%	25	14.45%	173	9.29%
Hitting an object	51	62.20%	31	37.80%	82	4.40%
*Truck characteristics*						
*Truck type*						
Light truck *	480	60.61%	312	39.39%	792	42.53%
Medium truck	285	64.33%	158	35.67%	443	23.79%
Heavy truck	395	63.00%	232	37.00%	627	33.67%
*Braking performance*						
Normal *	959	60.13%	636	39.87%	1595	85.66%
Impaired	201	75.28%	66	24.72%	267	14.34%
*Temporal characteristics*						
*Season*						
Spring	326	64.30%	181	35.70%	507	27.23%
Summer *	231	56.07%	159	38.59%	412	22.13%
Autumn	256	61.69%	181	43.61%	415	22.29%
Winter	347	65.72%	181	34.28%	528	28.36%
*Time*						
Night	115	66.86%	57	33.14%	172	9.24%
Morning	316	62.08%	93	18.27%	509	27.34%
Afternoon *	420	60.00%	280	40.00%	700	37.59%
Evening	309	64.24%	172	35.76%	481	25.83%

* The variable serves as a reference.

**Table 2:** Estimated results for the binary logit model and the random parameter logit model.

Variables\Items	Binary Logit Model			Random Parameter Logit Model		
	Coefficient	Std. Err.	$p>\left|z\right|\;$	Coefficient	Std. Err.	$p>\left|z\right|\;$
*Roadway characteristics*						
*Highway classification*						
Freeway	0.635	0.211	0.003	−0.733 (0.900)	0.186	<0.001
Provincial highway	−0.539	0.213	0.011	−0.520	0.179	0.004
Country road	−0.580	0.175	0.001	−0.623	0.148	<0.001
Urban road	−0.380	0.206	0.065	−0.491	0.175	0.005
Urban expressway	−0.433	0.494	n.s. ^a^	−0.368	0.452	n.s.
Other types of roads	−0.299	0.252	n.s.	−0.166	0.214	n.s.
*Horizontal alignment*						
Single curve	0.387	0.140	0.006	0.3954	0.118	0.001
Consecutive curve	−0.068	0.723	n.s.	−0.195	0.692	n.s.
*Pavement material*						
Gravel	0.242	0.338	n.s.	0.190	0.281	n.s.
Cement	−0.398	0.138	0.004	−0.364	0.116	0.002
Dirt	1.358	0.761	0.074	1.149	0.623	0.065
*Road surface condition*						
Wet	−0.365	0.182	0.045	−0.465	0.158	0.003
Other types of conditions	1.049	1.110	n.s.	0.719	0.795	n.s.
*Environmental characteristics*						
*Lighting condition*						
Daylight	0.145	0.240	n.s.	0.144	0.219	n.s.
Dark, not lit	−0.300	0.220	n.s.	−0.216	0.215	n.s.
*Accident characteristics*						
*Collision type*						
Head-on	−0.692	0.155	<0.001	−0.602	0.130	<0.001
Broadside	−0.908	0.192	<0.001	−0.877 (1.472)	0.171	<0.001
Rear-ending	−0.270	0.198	n.s.	−0.112	0.173	n.s.
Sideswipe	−0.385	0.251	n.s.	−0.381	0.211	0.071
Rollover	0.751	0.260	0.004	0.693	0.215	0.001
Hitting an object	−0.464	0.276	0.093	−0.525	0.231	0.023
*Vehicle characteristics*						
*Truck type*						
Medium truck	0.156	0.130	n.s.	0.099	0.110	n.s.
Heavy truck	0.114	0.120	n.s.	0.142	0.101	n.s.
*Braking performance*						
Impaired	0.581	0.164	0.000	0.617 (0.657)	0.146	<0.001
*Temporal characteristics*						
*Season*						
Spring	0.268	0.144	0.063	0.556 (2.538)	0.136	<0.001
Autumn	0.303	0.150	0.043	0.321	0.121	0.008
Winter	0.438	0.142	0.002	0.419	0.115	<0.001
*Time*						
Night	0.421	0.268	n.s.	0.439	0.236	0.063
Morning	0.118	0.127	n.s.	0.148	0.102	n.s.
Evening	0.422	0.243	0.083	0.873 (3.369)	0.236	<0.001
*Model statistics*						
Number of observations	1862			1862		
Log-likelihood at zero	−1233.730			−1151.216		
Log-likelihood at convergence	−1151.216			−1144.624		
AIC	2364.4			2362.6		

^a ^n.s. refers to non-significant (at the 90% confidence level).

**Table 3:** Marginal effects for the random parameter logit model.

Variables\Items	Marginal Effects
	Death
*Roadway characteristics*	
*Highway classification*	
Freeway	−0.165
Provincial highway	−0.117
Country road	−0.140
Urban road	−0.110
Urban expressway	−0.083
Other types of roads	−0.037
*Horizontal alignment*	
Single curve	0.089
Consecutive curve	−0.044
*Pavement material*	
Gravel	0.043
Cement	−0.082
Dirt	0.258
*Road surface condition*	
Wet	−0.105
Other types of conditions	0.162
*Environmental characteristics*	
*Lighting condition*	
Daylight	0.032
Dark, not lit	−0.049
*Accident characteristics*	
*Collision type*	
Head-on	−0.135
Broadside	−0.197
Rear-ending	−0.025
Sideswipe	−0.086
Rollover	0.156
Hitting an object	−0.118
*Temporal characteristics*	
*Truck type*	
Medium truck	0.022
Heavy truck	0.032
*Braking performance*	
Impaired	0.139
*Temporal characteristics*	
*Season*	
Spring	0.125
Autumn	0.072
Winter	0.094
*Time*	
Night	0.099
Morning	0.033
Evening	0.196

## Data Availability

The data presented in this study are available on request from the corresponding author. The data are not publicly available due to the drivers’ privacy.
